# Disordered eating and type 2 diabetes: The case for T2DE

**DOI:** 10.1111/dme.70150

**Published:** 2025-10-03

**Authors:** Anthony P. Winston, Alexandra Eriksson

**Affiliations:** ^1^ Clinic for Eating Disorders and Diabetes Coventry and Warwickshire Partnership Trust Warwick UK; ^2^ University of Warwick Coventry UK

**Keywords:** binge eating disorder, diabetes mellitus, feeding and eating disorders, type 2 diabetes

The combination of an eating disorder and diabetes poses unique therapeutic challenges. Recently, there has been an increase in both clinical and research interest in disordered eating in type 1 diabetes, which is now referred to as ‘T1DE’, although services for this condition remain limited. However, there has been no comparable increased interest in disordered eating in type 2 diabetes (T2DE). This commentary argues the case for the recognition of T2DE as an important condition, which warrants more attention.

Eating disorders have a prevalence of up to 20% in people with type 2 diabetes, and binge eating disorder (BED) is by far the most common diagnosis.^1^ A systematic review found a point prevalence of BED in adults with type 2 diabetes of between 1.2% and 8.0%.^2^ BED is the least well‐known of the eating disorders and often goes undiagnosed and untreated. Its defining feature is binge eating, without the use of compensatory behaviours such as vomiting, periods of starvation, excessive exercise or laxative misuse that are seen in bulimia nervosa. The American Psychiatric Association (*Diagnostic and Statistical Manual of Mental Disorders*, 5th Edition) and the World Health Organization (*International Classification of Diseases*, 11th Revision) have published similar diagnostic criteria for BED. There are a number of screening tests for BED, such as the Binge Eating Scale and the Binge Eating Disorder Screener–7, but none has been validated for use in people with T2DE.

Given the much greater prevalence of type 2 diabetes compared to type 1 diabetes, it is likely that a significant number of people with disordered eating and type 2 diabetes are not receiving appropriate help. Diabetes UK estimates that there are 4.4 million people living with diabetes in the United Kingdom.^3^ Assuming, conservatively, that 85% have type 2 diabetes and 5% of these have an eating disorder, there will be at least 187,000 people in the United Kingdom with T2DE.

BED exacerbates type 2 diabetes, both directly through the consumption of large amounts of carbohydrate and indirectly through the promotion of obesity. People with BED are estimated to be 3–6 times more likely to have obesity than those who do not have an eating disorder.^4^ Among people with type 2 diabetes, co‐morbid eating disorders are associated with higher HbA1c, which seems to be mediated primarily by higher BMI.^1^, ^2^, ^5^ Clinical experience suggests that many people with BED and type 2 diabetes are affected by weight stigma and may avoid contact with diabetes services due to embarrassment about their weight or diabetes self‐management.

The National Institute for Health and Care Excellence^6^ recommends eating disorder‐focused cognitive‐behavioural therapy (CBT‐ED) for the treatment of BED and this has been shown to reduce binge eating in 68–90% of participants, with total remission in around 50%.^6^, ^7^ There is increasing evidence for guided self‐help for less severe BED, which is based on a written CBT manual supplemented by a small number of sessions with a clinician.^7^ However, CBT‐ED alone has little effect on weight and it is at present unclear what effect it has on blood sugar in people with type 2 diabetes.^7^ It is uncertain whether standard CBT‐ED is effective in T2DE or whether it needs to be modified to take into account the complex relationships between eating and diabetes. An online‐guided self‐help programme for T2DE (POSE‐D) is currently in development.^8^

Drug treatment has a limited role in the treatment of BED. No medication is licensed for the treatment of BED in the United Kingdom. In the United States, the only licensed drug is lisdexamfetamine, which has been shown to be superior to placebo in reducing both bingeing and weight over the short term; however, its long‐term effectiveness is unknown.^9^ Topiramate has also shown positive short‐term effects on both bingeing and weight in clinical trials but has frequent side effects.^9^ The role of glucagon‐like peptide‐1 receptor agonists (GLP‐1 RAs) and glucose‐dependent insulinotropic polypeptide receptor agonists is controversial. There is some evidence that GLP‐1 RAs reduce binge eating but their long‐term effects on BED are unknown and there is concern that they may cause excessive weight loss and the development of a restrictive eating pattern.^10^

Calorie, and particularly carbohydrate, restriction is thought to worsen binge eating, and a regular intake of carbohydrate is often recommended in the treatment of BED. However, this conflicts with the advice often given to people with type 2 diabetes and obesity, and this can be difficult for people with T2DE to manage. It is therefore important that the management of T2DE is carried out by professionals with knowledge of both eating disorders and diabetes.

Recognition of T2DE presents an opportunity to develop novel, targeted interventions which will improve outcomes in terms of both diabetes and mental health. More research is needed into effective treatments, and specific screening tools need to be developed and implemented. More specialist services are required, with expertise in both eating disorders and diabetes. Most importantly, healthcare professionals need to be made more aware of T2DE. Use of the acronym ‘T1DE’ (pronounced ‘tied’) has increased awareness of disordered eating in type 1 diabetes; promotion of the acronym ‘T2DE’ (perhaps pronounced ‘tood’) might bring similar benefits in type 2 diabetes.

## FUNDING INFORMATION

None.

## CONFLICT OF INTEREST STATEMENT

The authors declare no conflicts of interest.
